# Supplementary material for: Patterns of Intron Gain and Loss in Fungi
Source: PLoS Biol. 2004 Nov 30;2(12):e422. doi: 10.1371/journal.pbio.0020422 (PMC532390; doi:10.1371/journal.pbio.0020422)
Supplement: Table S1 — Also available at http://genes.mit.edu/NielsenEtAl/. (4.3 MB ZIP). [file pbio.0020422.st001.zip › NielsenEtAl/html/1346.html]

AN6502.1.NCU04180.1.MG03974.1.FG07341.1


```
 CLUSTAL W (1.82) Multiple Sequence Alignments - Introns Inserted


Sequence 1: NCU04180.1	351 aa
Sequence 2: MG03974.1	356 aa
Sequence 3: FG07341.1	370 aa
Sequence 4: AN6502.1	328 aa
Alignment Length: 440 aa
Number Identitical Residues: 89 aa
Alignment Score (without introns) 5697


MG03974.1 	---------~----------------~-------------~------------~------
NCU04180.1	---------~----------------~-------------~------------~------
FG07341.1 	MFALSEESK0ERIAKLIDVSRVAIHY2YRSGYLPLILYLG1YTRSEPRPSVIR2DVGFMR
AN6502.1  	---------~----------------~-------------~------------~------
          	                                                            

MG03974.1 	----~---MVAANSVGR~DGLSPAM-VETVAG--~------LSAG~TAAT~LAVHPLDIV
NCU04180.1	----~---MTKSSDAG-~--LSPAL-VETVAG--~------LSAG~SMAT~LIVHPLDIV
FG07341.1 	QKAS0FFFMPDFNNAG-~--LSPAT-IESIAG--~------LSAG~TVST~LTVHPLDVV
AN6502.1  	----~---MVQFSEETK0ERISKVIDVSRVAVHY2GYLPLILYLG1SSDI1QPLCPSGYN
          	        *   ..    . :* . .:. :*    .  .  *  * :      : * .  

MG03974.1 	KTRMQI1HRSNAAAAASASSKPGLAQQPPPLRAVAVLRSLVQTEKPIAALYRGLTPNLVG
NCU04180.1	KTRMQV1HRSSPTNPSAA------------LTTVSVFRSLAQTDRPLAALYRGLTPNLIG
FG07341.1 	KTRMQI1YRSTAPGAVRP-------------TTVSILRALTSTPHPIASLYRGLTPNLVG
AN6502.1  	TSNIRL~YTVDRTSSSRVG------------VSLRVIREIFHKEGGLIAFYRGLTPNLIG
          	.:.::: :    . .   .             :: ::* :  .   : ::********:*

MG03974.1 	NATSWASFFFFKKRCEQAILSLKKPQ-PAADGSL----------VVNDGDKYDDGSARLT
NCU04180.1	NATSWASFFFFKSRFERLIAHLKAPSSPPAPSHLGGEPTTLPQNVTPDRETQAQIKSHLS
FG07341.1 	NASSWASFFFFKSRFENTIAAWQGRP-----------------------------DGRPT
AN6502.1  	NSSSWALYFLFYDNVKEILGSWRSRS---------------------NSNGSQQRREPLE
          	*::*** :*:* .. :. :   :                        . .   .      

MG03974.1 	PQDYFVSSAAAG~AAVQVLTNPVWVLKTRMLSSDRGSVGAYPSMWVGATRVWREDGPRGF
NCU04180.1	PTDFFAASLLAG~AATQIITNPIWVLKTRMLSTDRLAADAYPSMFTGAVRLFRSEGILGF
FG07341.1 	PGDYFVASALAG~ASTTTLTNPIWVLKVRMVSSDRGSRGAYPSMLAGARSILQTEGIRGF
AN6502.1  	ASDYFIASGSAG1IITSILTNPIWVIKTRMLATGSMSPGAYTSFTAGAMQILRSEGVPGF
          	. *:* :*  **   .  :***:**:*.**:::.  : .**.*: .**  : : :*  **

MG03974.1 	YRGLGISLLGVSHGAVQFAVYEPAKRLYVVWRSRGRQHDAASAAKPAAGGS-VMTNEATL
NCU04180.1	YRGLGVGMLAISHGAVQFAVYDPARRMYIASRDAKRRLAGQEIASEDERESQRISNEATI
FG07341.1 	YRGLGISLVGVSHGAVQFAVYDPMKRLYHARR---REKYGLERDH--------MTTEATI
AN6502.1  	YRGLVPSLFGVSHGALQFMAYEKLK--FHRAN---AHSGGLQRKE--------LSNMDFF
          	****  .:..:****:** .*:  :  :   .    .  . .           ::.   :

MG03974.1 	VISTAAKLFANAVTYPYQVLRSRLQNYDAENQFGRGIVGAATRLWREEGVRGFYRGLVPG
NCU04180.1	VLSTAAKLVAGTATYPLQVMRARLQHHQADELFGRGIGGVVAKLWREEGFRGFYRGMMPG
FG07341.1 	GLSSLAKFVAGAVTYPYQVLRSRLQNYEADKRFGRGIRGAVVRIWTEDGLRGFYRGLVPG
AN6502.1  	IISSVSKIFAGSITYPYQVLRSRLQTYDAYLAY-RGLQDAIVKIWATEGLGGFYKGLGPN
          	 :*: :*:.*.: *** **:*:*** ::*   : **: .. .::*  :*. ***:*: *.

MG03974.1 	VVRVLPATWVTFLVYENTKYYLPRWTAGSQA
NCU04180.1	VVRVLPATWVTFLVYENVKYYLPKMVSGV--
FG07341.1 	VVRVMPATWVTFLVYENVKYYIPYWV-----
AN6502.1  	LFRVLPSTWVTFLMYENTRAYLSKVMSNA--
          	:.**:*:******:***.: *:.   :.
```
